# Protocol for a scoping review to map evidence from randomised controlled trials on paediatric eye disease to disease burden

**DOI:** 10.1186/s13643-017-0564-x

**Published:** 2017-08-18

**Authors:** Lucinda J Teoh, Ameenat L Solebo, Jugnoo S Rahi

**Affiliations:** 1 Lifecourse Epidemiology and Biostatistics Section, Population, Policy and Practice Programme, UCL Great Ormond Street Institute of Child Health, 30 Guilford Street, London, WC1N 1EH UK; 2 Great Ormond Street Hospital for Children, NHS Foundation Trust, London, UK; 3 Ulverscroft Vision Research Group, London, UK; 4 Moorfields Eye Hospital, NHS Foundation Trust/NIHR Moorfields Biomedical Research Centre, London, UK

**Keywords:** Childhood, Blindness, Vision disorders, Epidemiology, RCTs, Burden of disease

## Abstract

**Background:**

Currently, about 2 per 1000 children in the industrialised world are severely visually impaired or blind (SVI/BL) due to diverse uncommon conditions that are usually present from early infancy. The impact of SVI/BL is lifelong and life-changing. Thus, children are a priority in the WHO-led global initiative against avoidable blindness. The aim of this scoping review is to assess the current evidence base on interventions to prevent or treat the major causes of childhood SVI/BL, specifically the degree of alignment between robust interventional research (RCTs) and the burden (relative frequency) of the key causative disorders, identifying gaps in the evidence base for tackling childhood blindness.

**Methods/design:**

We will perform a scoping review of the published literature of randomised controlled trials (RCTs) for clinical interventions that prevent or treat eye and vision diseases in children (<18 years old). Major electronic databases MEDLINE (PUBMED), EMBASE and the Cochrane CENTRAL will be searched to identify published trials using a comprehensive paediatric specific strategy informed by previous searches. The outcome of our study, randomised clinical trial activity, will be measured by the total number of RCTs and total paediatric participants randomised. The quantity and distribution of activity across diseases will be classified in the broad categories of anatomical site affected (per WHO taxonomy). The degree of alignment between paediatric trial activity and burden of SVI/BL disease (relative proportion) will be measured using a test of association (Spearman’s correlation coefficient).

**Discussion:**

Despite the global public health importance of childhood blindness, there has been no assessment of the completeness of the evidence base regarding clinical interventions to prevent or treat the causative disorders. This scoping review will measure the degree of alignment between the published evidence and the burden of disease to identify gaps in current knowledge and consider the underlying reasons, informing clinicians, policy makers and funders about research priorities.

**Electronic supplementary material:**

The online version of this article (doi:10.1186/s13643-017-0564-x) contains supplementary material, which is available to authorized users.

## Background

### The importance of paediatric eye disease

Paediatric eye and vision disorders are common, but most are self-limiting or are easily fully addressed (e.g. spectacles for refractive error) or amenable to good visual outcomes after treatment: for example, amblyopia (“lazy eye”) affecting one eye occurs in between 1 and 7% of young children [1–7], and good improvements in vision are achievable with treatment. In contrast, visual impairment (VI) and severe visual impairment/blindness (SVI/BL) are uncommon in the industrialised world affecting about 2 per 1000 children, usually from birth or infancy. VI and SVI/BL confers lifelong and life-changing consequences for affected individuals, their families and the societies in which they live, for example, through the significant impact on development and learning [8–10].

The global burden of disease is conventionally measured using DALYs (disability-adjusted life years), but the lack of data on children, reflecting a paucity of national epidemiological studies of visual impairment [11], means the burden of childhood blindness has to be inferred from the YLD (years lived with disability) component of DALYs, which is higher compared to adults, because it occurs so much earlier (on average 7 decades earlier) in the life course. Thus, children are a priority in VISION 2020, the WHO-led global initiative against avoidable blindness [8, 12].

Severe visual impairment/ blindness (SVI/BL) in children is caused by a heterogeneous group of disorders which are individually uncommon. From the only national epidemiological study of incident childhood SVI/BL, which was carried out in the UK in 2000, it is estimated that in high-income countries, 4 in 10,000 children will become severely visually impaired or blind before their first birthday, with that number increasing to 6 in 10,000 before the age of 16 [9]; from that study, the relative frequency of different underlying disorders causing SVI/BL is known. Directly comparable information for middle- and low-income settings are not available, but other studies indicate a significantly greater rate and a very different pattern of causes [10]. Findings from an on-going national study of childhood visual impairment (VI) as well as SVI/BL, i.e. *full spectrum* visual disability (acuity 0.5 LogMAR or worse in the better eye) in the UK, will provide data on incidence and the current pattern of causes [13].

Despite the global public health importance of childhood blindness, there has not yet been a study that maps the completeness or coverage of the current evidence base on interventions to prevent or treat the major causes of childhood SVI/BL. The aim of this scoping review is to measure the distribution of robust interventional research (RCTs) across paediatric eye disease and to determine the association between the quantity of good quality RCTs and the burden of disease (relative frequency) of the key causative disorders, identifying gaps in the evidence base for tackling childhood blindness and considering the reasons for this, so as to inform research strategies and priorities.

The review will address the following questions in relation to eye and vision disease in children:

1. How many randomised controlled trials in children have been published and how many children/young people have participated?
2. What is the distribution of research activity (the ‘quantity’ of RCT evidence) by disease, distinguishing disorders associated with SVI/BL from those that do not cause visual impairment?
3. What is the distribution of research activity by disease as classified by anatomical site affected and sub-classified by disorder
4. How does the distribution of research activity by disease correlate with burden of childhood SVI/BL, as classified by comparative incidence of SVI/BL by anatomical site affected?
5. What are the gaps in our knowledge base in terms of interventions for childhood vision and eye disease?

## Methods

### Study design

This scoping review followed the framework outlined by Arksey and O’Malley [14] and also adopted the updated recommendations by Levac and colleagues for scoping of the literature [15, 16]. In addition, this review will incorporate an evidence map to provide a user-friendly format to illustrate the evidence gaps that exist in terms of interventions to address severe visual impairment and blindness in children. To ensure that the evidence map produced is useful and easily interpretable to stakeholders, the framework suggested by Bragge and colleagues produced for the global evidence mapping initiative was followed [17, 18].

Our scoping review protocol will use a systematic approach to searching the literature for randomised controlled trials in the broad topic area of interventions to prevent or treat paediatric eye disease. The PRISMA-P and PRISMA-PC statements were used to guide the reporting of this protocol [19–21]. The populated PRISMA-P checklist is available as a supplementary file to this protocol (Additional file 1). In addition to descriptive data extracted from each trial regarding the disease investigated, intervention type and type of outcome measures, we will focus on two key data points: the number (quantity) of trials (per disease) and the number of children/young people randomised. These two indicators of clinical research activity will be mapped to the burden of disease for each blinding condition in order to understand the distribution of robust evidence across the breadth of paediatric eye disease.

### Eligibility criteria

The following inclusion criteria will be used to guide the search and will form the basis of the inclusion criteria at the title and abstract level:

*Participants*: any trials where children (of 0 to 18 years of age, in line with Article 1 of the United Nations Convention on the Rights of the Child [22]) comprise the total or majority of the study population. This was defined as a trial that included exclusively participants aged within 0–18 years or had a mean or median age of < 18 years if the trial included adult participants. Aside from age, there were no further restrictions on characteristics such as gender or ethnicity of participants.

*Types of studies*: Only randomised controlled trials reporting results will be included. Quasi-RCTs will be excluded. Protocols for randomised controlled trials will be excluded. There will be no restriction with regards to the date of publication, country context of the trials, language or publication status.

### Search strategy

In order to maintain sufficient breadth in our search to capture all eye and vision disorders, we will use general search terms (such as the MeSH terms ‘Eye diseases’ and ‘Eye’) to cover all major indexed eye conditions. As these terms are automatically exploded in MEDLINE, it is deemed broad enough to include all ophthalmic conditions of interest. RCTs of conditions that are not associated with bilateral visual impairment (e.g. conjunctivitis, blepharitis, amblyopia, squint) will be enumerated separately to provide context and scale for the RCT evidence in relation to disorders that cause SVI/BL. We will limit the scope of this search to include only randomised controlled trials as this study focuses on the most robust evidence from interventional research that is used to inform clinical decisions and policy making.

Preliminary searches were carried out and the strategy refined after reviewing the relevance of preliminary results. One example of an adjustment to our search strategy was in response to many studies that were included as they mentioned RCTs in their abstracts but were not themselves a randomised controlled trial in their study design. In response to this finding, we removed the following publication types using the Boolean NOT term: reviews, meta-analyses, practice guidelines, observational studies, editorials, comments and letters. We also adjusted the paediatric filter within our search so that all paediatric specific terms had to be present in the title or abstract, after noting that many studies that do not include children but includes participants 18 years and over were indexed with the ‘Adolescent’ MeSH term (defined by PUBMED as ages 13–18 years old).

### Search methods

**Fig. 1:**
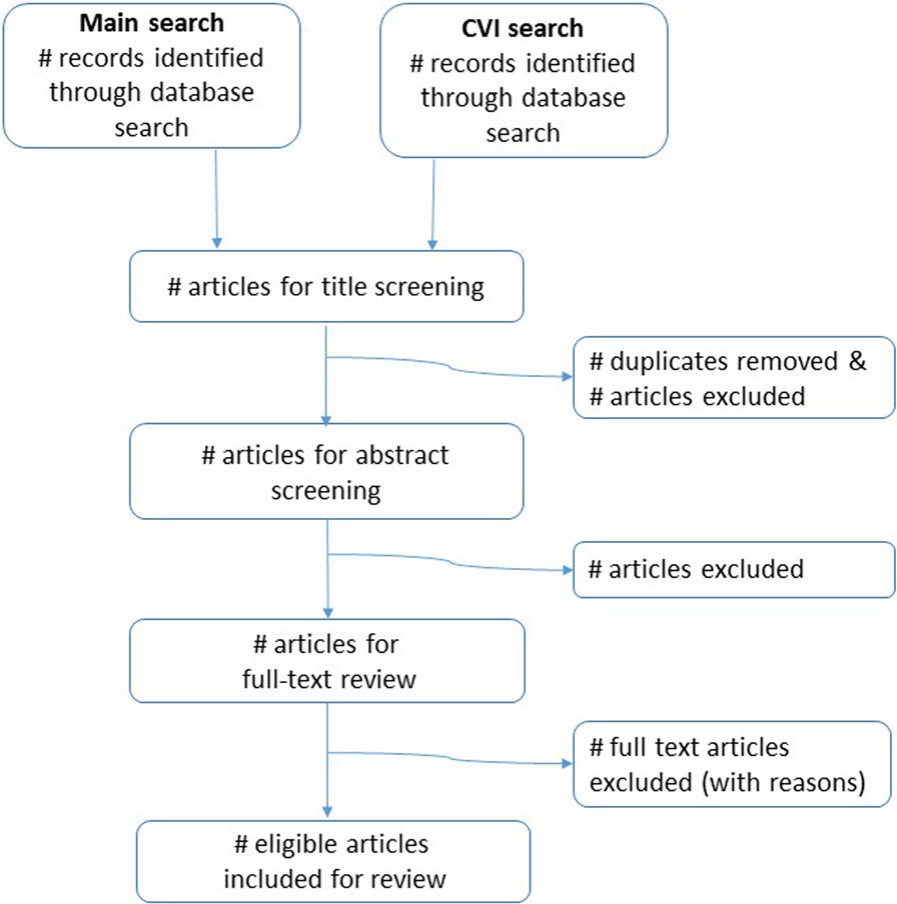
Proposed search strategy and screening process

MEDLINE (PUBMED), EMBASE and CENTRAL in the Cochrane Library will be searched for randomised controlled trials reporting results. In order to maximise the comprehensiveness of the search, there will be no restriction on date of publication. Paediatric search terms created by authors will incorporate terms from the validated *child filter* for MEDLINE [23]. The MEDLINE search strategy will be adapted to suit EMBASE (Ovid interface). A second search will be carried out to target trials addressing the cerebral causes of SVI/BL (known as *cerebral visual impairment* or *cortical visual impairment* (CVI)) that are not necessarily indexed as ‘eye disease’ and thus not captured in the first search. The CVI search strategy has been developed in an iterative manner, based on indexed search terms used in publications of known trials measuring CVI as an outcome measure (e.g. the Cool-cap trial [24]). This two-pronged search strategy is illustrated in Fig. 1. The entire search strategy is available as a supplementary file to this protocol (see Additional file 2). Reference lists from eligible studies will also be checked to ensure no pertinent trials are missed.

### Study selection

Records from all databases will be exported into and managed in EndNote × 7 referencing software. Following the removal of duplicates, eligibility assessment will be performed independently by two reviewers (LJT and ALS). Double counting of trials will be avoided by synthesising data from multiple reports using the trial registration number, where available, to identify unique trials. The reason for trial exclusion will be recorded, e.g. not a randomised trial (non-RCT), vision disorder not a primary disease of interest, or trial does not include children. Any disagreement between reviewers will be resolved by consensus. The senior author (JR) will hold a determining vote if the consensus is not reached. Dual data extraction for both clinical trial activity measures will be carried out independently by LJT and ALS. Full data extraction from trials will be carried out by LJT. ALS will independently extract the full dataset from a random sample of 33% of eligible trials. If there is more than 10% disagreement on collected data, the second reviewer (ALS) will extract the full dataset for all eligible titles.

### Risk of bias

Two reviewers (LJT and ALS) will independently assess the methodological quality across eligible trials using the Cochrane collaboration’s risk of bias tool for randomised trials [25], which specifies six bias domains at the study level that will be independently assessed by judgement of both reviewers. The level of overall bias across the six domains will be reported across trials as a low, unclear or high risk of bias with support for the judgement provided as per the recommended list of items. Any disagreement between authors over the risk of bias of a particular publication will be resolved by discussion with a third independent reviewer (senior author).

### Classification of disorders

Disorders studied in RCTs will be classified as either potentially causing SVI/BL or not independently by the authors (LJT, ALS, JSR) and any disagreements resolved by consensus.

### Charting the data

Ten key data items will be extracted from eligible trials and tabulated in a spreadsheet including one of the review measures of clinical trial activity, i.e. the number of children included in each trial (by disease). The list of items extracted from each trial will also be presented collectively in evidence tables, with trials categorized into one of eight groups based on the anatomical site of injury: *visual pathways and cortex*; *whole globe and anterior segment*; *cornea*, *lens*, *uvea*, *retina*, *optic nerve*, *nystagmus* and *other*.

The following data will be extracted from each eligible trial:

1. Bibliometrics: Authors, publication year, title, journal
2. Type of RCT (if specified)
3. Method of randomisation
4. Sample size of paediatric participants randomised
5. Paediatric age range
6. Disease type (anatomical classification)
7. Intervention type (pharmacological, dietary supplement, procedure, device or other) and description
8. Comparator intervention
9. Primary ophthalmic outcome measure (or secondary outcome measure for CVI trials)
10. Length of follow-up

### Burden of paediatric eye disease

The relative frequency (%) of each disorder causing SVI/BL in children (based on the annual incidence as reported in the national surveillance study of SVI/BL in the UK [9]) will be used as the proportional measure of burden for each eye condition in the absence of global burden of disease data in children. RCTs will be grouped according to the primary anatomical site affected (e.g. retina for retinopathy of prematurity) in order to assess the distribution of evidence across all SVI/BL diseases, as per the WHO taxonomy for causes of childhood blindness.

### Collating, summarising and reporting the results

The extent of the evidence from RCTs for each disorder that causes SVI/BL will be measured in two ways: the total number of RCTs involving children/young people and total number of paediatric participants randomised. Histograms will be used to plot these two measures of clinical trial activity grouped by anatomical site. Scatter plots will present total number of RCTs and total paediatric participants (clinical trial activity) by the burden (relative frequency of disease). The degree of alignment between trial activity and burden which will be measured using an appropriate test of association will be carried out and reported (Spearman’s correlation coefficient).

### Evidence map

A bubble chart will be used to present visually the quantity of trial evidence (total RCTs) mapped against the burden of disease (relative frequency of disease), with the size of each bubble proportional to the total number of paediatric participants. Each bubble will be coded to identify the anatomical site affected.

## Discussion

The intensity and focus of randomised trial research should ideally align with the global burden of disease. Measuring this association is important, as clinicians, funding bodies and policy makers should be aware of the areas where evidence is lacking in order to actively adjust research priorities. Children with SVI/BL are a clinically heterogeneous population and require targeted research to cover the many and multiple disorders that lead to early visual impairment or blindness.

A poor association has been reported between the quantity of randomised controlled trials and the global burden of disease [26], specifically with only a moderate association reported for RCT activity in paediatrics and child health [27]. No study has investigated this question of the association between the quantity and distribution of RCT evidence on interventions and the burden of disorders that cause visual disability. Therefore, the unique aim of this scoping review is to ascertain whether randomised trials of interventions to prevent or treat eye and vision disorders that cause SVI/BL in children actually reflect the burden of disease in industrialised countries for whom the necessary data on burden of disease are available.

We will use the findings of this review to highlight any mismatch between the burden of childhood eye diseases and the evidence, focusing specifically on the blinding disorders that confer the highest impact. In identifying these gaps in our knowledge with regards to interventions, we will also discuss the potential barriers that have led to this disparity. Additionally, this review will provide a unique summary of all the interventions and visual outcome measures used in RCTs in all ophthalmic disorders in childhood, including common conditions that do not confer visual impairment.

## Additional files

Additional file 1: The guidelines outlined in the PRISMA-P checklist were followed for this protocol, and the location of each point is illustrated in the attached table in Additional file 1. (DOCX 32 kb)

Additional file 2: A complete list of search terms used for search in all literature databases including PUBMED, EMBASE and Cochrane CENTRAL is included in Additional file 2. (PDF 255 kb)

## Abbreviations

DALYs: Disability-adjusted life years
PRISMA-P: Preferred Reporting Items for Systematic Reviews and Meta-Analysis Protocols
PRISMA-PC: Preferred Reporting Items for Systematic Reviews and Meta-Analysis Protocols in Newborns, Children, and Adolescents
RCT: Randomised controlled trial
SVI/BL: Severe visual impairment or blindness
WHO: World Health Organization
