# Supplementary material for: Protocol for a scoping review to map evidence from randomised controlled trials on paediatric eye disease to disease burden
Source: Syst Rev. 2017 Aug 18;6:166. doi: 10.1186/s13643-017-0564-x (PMC5563052; doi:10.1186/s13643-017-0564-x)
Supplement: Supplementary file 2 — A complete list of search terms used for search in all literature databases including PUBMED, EMBASE and Cochrane CENTRAL is included in Additional file 2. (PDF 255 kb) [file 13643_2017_564_MOESM2_ESM.pdf]

Additional file 2: Full search strategy

a. **MEDLINE (PUBMED) search strategy for all paediatric eye diseases**

1. random*.ti,ab.
2. trial.ti,ab.
3. 1 and 2
4. randomized controlled trial.pt
5. 4 and 3
6. randomized controlled trial/
7. 6 and 3
8. 3 or 5 or 7
9. not randomized controlled trials/ (as major topic)
10. not Review.pt
11. not Meta-analysis.pt
12. not Practice Guideline.pt
13. not Observational study.pt
14. not Editorial.pt
15. not Comment.pt
16. not Letter.pt
17. and/9-16
18. not “protocol”.ti
19. not “non-randomized”.ti,ab
20. not “non randomized”.ti,ab
21. 17 and 18 and 19 and 20
22. 8 and 21
23. Eye disease/
24. Vision, Ocular/
25. “visual impairment”.ti,ab.
26. “childhood blindness”.ti,ab.
27. or/23-26
28. Child*.ti,ab
29. Infant*.ti,ab
30. Newborn*.ti,ab
31. Baby.ti,ab

32. Neonat*.ti,ab
33. Preterm*.ti,ab
34. Prematur*.ti,ab
35. Schoolchild*.ti,ab
36. Preschool.ti,ab
37. Kindergar*.ti,ab
38. Kid*.ti,ab
39. Toddler*.ti,ab
40. Adolesc*.ti,ab
41. Teen*.ti,ab
42. Pubert*.ti,ab
43. or/28-42
44. 22 and 27 and 43

b. MEDLINE (PUBMED) search strategy for cerebral visual impairment (CVI)

1. hypoxia-ischemia, brain/
2. encephalopathy.ti,ab.
3. brain ischemia/
4. or/1-3
5. cortical adj visual adj2 impairment.tw
6. cortical adj blindness.tw
7. cerebral blindness.tw
8. cortical blindness/
9. visual impairment/
10. low vision/
11. developmental disabilities/
12. neurodevelopmental adj disability.ti,ab.
13. neurodevelopmental adj outcome.ti.ab
14. vision disorder/
15. or/5-13
16. randomized controlled trial/
17. randomized control* AND trial.ti.ab
18. randomized clinical trial.ti,ab.

19. randomized trial.ti,ab

20. randomized controlled trial.pt

21. or/16-19

22 newborn infant/

23 infant/

24 preterm birth/

25 infant, low birth weight/

26 or/21-24

27 4 and 15 and 20 and 25

c. EMBASE (Ovid) search strategy for main search

1. random*.ti,ab.
2. trial.ti,ab.
3. 1 and 2
4. randomized controlled trial/
5. 3 or 4
6. Not conference abstract.pt
7. Not conference paper pt.
8. Not short survey.pt
9. Not meta-analysis/
10. Not non-randomized trial/
11. Not editorial/
12. Not observational study/
13. Not letter/
14. Not review
15. Not practice guidelines
16. Not “protocol”.ti
17. Not “non-randomized”.ti
18. Not “non randomized”.ab
19. and/6-18
20. 5 and 20
21. Eye diseases/
22. Vision, Ocular/

23. “visual impairment”.ab.
24. “childhood blindness”.ab.
25. visual impairment”.ti
26. “childhood blindness”.ti
27. Exp Vision Disorders/
28. Exp Eye abnormalities/
29. Retinal diseases/
30. Scleral diseases/
31. Uveal diseases/
32. Optic Nerve diseases/
33. Lens diseases/
34. Corneal diseases/
35. Eye neoplasms/
36. Eye infections/
37. or/21-36
38. Child/
39. Child*.ti,ab
40. Infant/
41. Newborn*
42. Baby*
43. Babies
44. Neonat*
45. Preterm*
46. Prematur*
47. Schoolchild*
48. Preschool*
49. Toddler*
50. Teen*
51. Adolesc*
52. Pediatrics/
53. Paediatric/
54. or/37-53
55. 20 and 37 and 53

d. Cochrane CENTRAL strategy for main search

All searched “in Trials”

1. Eye Diseases/
2. Child/
3. Infant/
4. Adolesc*
5. 2 or 3 or 4
6. randomized.ti,ab,kw
7. 1 and 5 and 6
